# METER2800: A novel dataset for music time signature detection

**DOI:** 10.1016/j.dib.2023.109736

**Published:** 2023-10-26

**Authors:** Jeremiah Abimbola, Daniel Kostrzewa, Paweł Kasprowski

**Affiliations:** Silesian University of Technology, Poland

**Keywords:** Time signature, Dataset, Meter

## Abstract

The Meter2800 dataset is an important contribution to Music Information Retrieval (MIR) research, as it is the first dataset to include audio files specifically designed for time signature detection. By combining audio files from three renowned datasets and including additional tracks, we have created a comprehensive and diverse collection of 2800 audio tracks that overcomes the limitations of existing audio datasets. The dataset includes 2.26GB of high-quality audio, which has been annotated with metadata, pre-computed features, tempo and time signature. In addition, we propose a train/test split and provide baseline results for time signature detection. The dataset is freely available for the research community and is available online for download. We believe that Meter2800 will contribute to the advancement of Music Information Retrieval research, particularly in the area of time signature detection. In technical validation, four classification experiments were conducted using four types of machine learning algorithms: SVM, KNN, Naive Bayes, and Random Forest.

Specifications Table

**Table:** 

Subject	Computer Science/Artificial Intelligence
Specific subject area	The Meter2800 dataset can be used to train AI models to detect music time signatures. This is an important task in music information retrieval (MIR), as it can help to improve the performance of music analysis and retrieval systems. AI models are trained on large datasets of labelled data. The Meter2800 dataset provides a rich source of labelled data for music time signature detection. It contains a diverse range of music genres and styles, which makes it well-suited for training robust AI models.
Data format	Raw (Audio files taken from other datasets) Annotated (Audio files analysed to get the time signature )
Type of data	csv files – annotation records tar.gz – zipped annotated files
Data collection	Meter2800 is a new dataset for music time signature detection, created by combining three popular MIR datasets (GTZAN, MagnaTagATune and FMA) and adding new audio files to augment for the insufficiencies of the other three. It is organized in a way that makes it easy for researchers to access and utilize. The creation process for the Meter2800 dataset involved several steps. First, a set of audio files was selected for analysis. The files were chosen to represent a diverse range of musical genres and styles. Next, every audio track of 30 seconds duration in the individual dataset was carefully analyzed by expert annotators. The annotators listened to each track multiple times to identify the time signature, which is the meter of the music. After identifying the time signature of each track, the annotators recorded this information along with other relevant metadata, such as the musical tempo. The tempo and the extracted features were processed using librosa; a library used for audio and music signal analysis in Python. Finally, the annotated data was cleaned and pre-processed to ensure that it was consistent and usable for further analysis. This involved removing any duplicates or errors in the data and converting it into a standardized format, such as CSV files.
Data source location	Music experts within the Department of Applied Informatics of the Silesian University of Technology
Data accessibility	Repository name: Harvard Dataverse Data doi : https://doi.org/10.7910/DVN/0CLXBQ Code doi: https://doi.org/10.6084/m9.figshare.24235396.v1 Direct URL to data: https://bit.ly/meter2800

## 1 Value of the Data

**Fig. 1:**
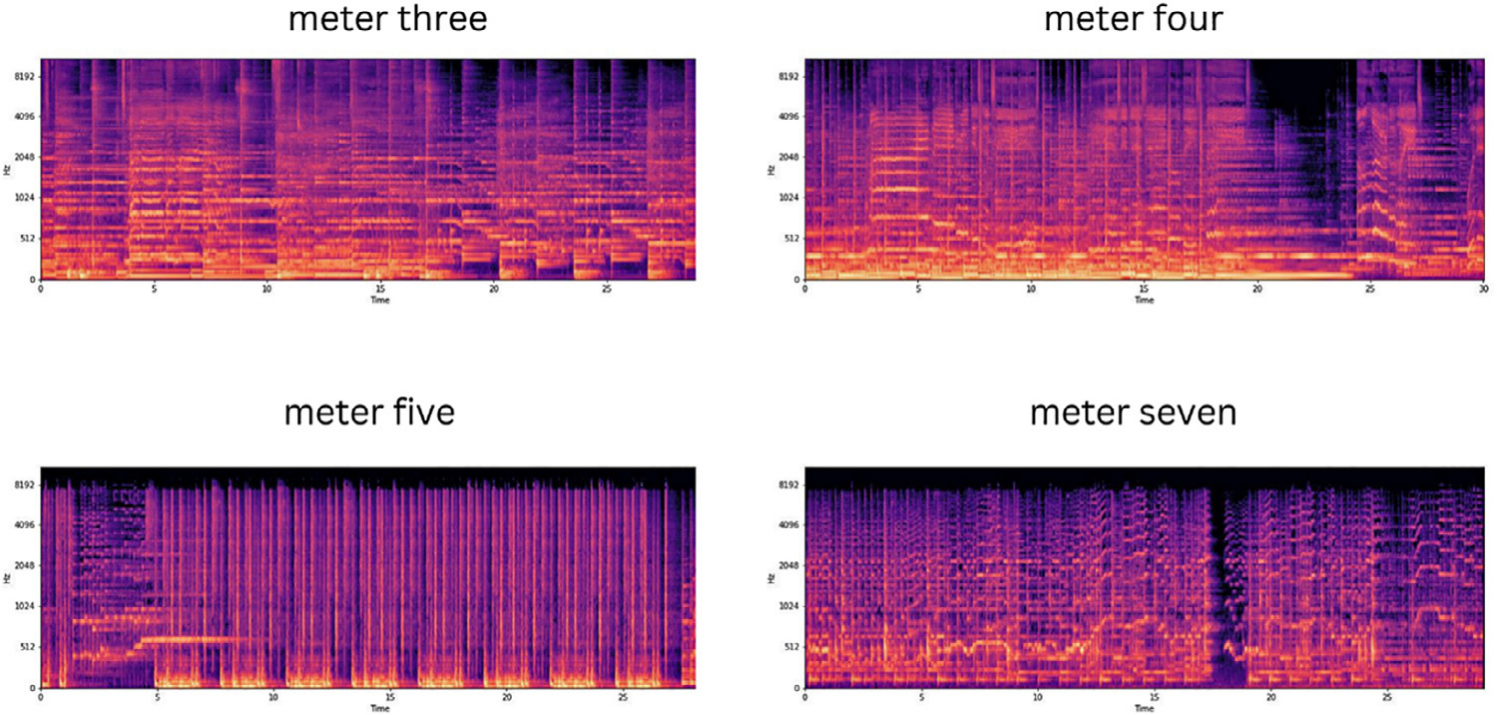
Sample spectrogram for one audio signal from each music meter class.

• This is the first-ever dataset that is specifically annotated with audio files for time signature detection. Prior to this dataset, there were no such resources available for MIR researchers to utilize. The only one that comes close is the Million Song Dataset (MSD) [1]. While it provides estimated time signatures, it should be noted that the meter values in the MSD were not directly annotated or labeled, but rather computed from the audio signals. As a result, these values may contain errors or inaccuracies. Furthermore, the MSD does not provide the actual audio files used for the time signature estimation.
• We believe that Meter2800 will contribute to the advancement of Music Information Retrieval research, particularly in the area of time signature detection and music classifications tasks.
• The audio files from each dataset are organized into separate folders in the Meter2800 dataset. The annotated data is split into train and test sets, each stored in separate CSV files.
• This dataset demonstrates the potential when used on machine learning models to classify audio files based on their meter values using extracted features such as spectrogram as shown in Fig. 1.

## 2 Data Description

Time signatures are crucial in notated music as they aid in organizing and measuring music, and segmenting it into phrases. It consists of two parts: a numerator indicating the number of beats per measure and a denominator indicating the note values of those beats. The denominator can be 4 for quarter notes, 2 for half notes, or 8 for eighth notes. The numerator is important for beat tracking, such as 2 for a simple duple meter of 2/4 and 6 for a complex/compound meter of 6/8. Our concentration is on the numerator of the meter since the denominator doesn't have anything to do with beat tracking.

The dataset is organized in a way that makes it easy for researchers to access and utilize. The audio files from each dataset are organized into separate folders in the Meter2800 dataset. This makes it easier for researchers to identify and extract audio files from specific datasets when needed. For instance, all the audio files from the GTZAN dataset are stored in a folder named "GTZAN," while the audio files from the FMA and MagnaTagATune datasets are stored in folders named "FMA" and "MAG," respectively. The additional audio file obtained from personal sources are put into the "OWN" folder.

The annotated data is split into train and test sets, each stored in separate CSV files. This makes it easy for researchers to use the dataset for training and testing machine learning models.

The dataset is accompanied by a detailed documentation file that provides information about the dataset's format, structure, and annotations. This documentation serves as a guide for researchers who want to use the dataset, providing them with detailed information about the characteristics, including the tempo, the number of segments for each sample, and the meter classes of each audio file (Table 1).

**Table 1:** Summary of data source and annotated files for Meter2800 dataset.

Data Source	Number of files Annotated	Train	Test
FMA	851	598	253
GTZAN	911	632	279
MAG	925	652	273
OWN	113	78	35

A more detailed description of the dataset is as follows:

• The dataset contains 2800 audio tracks, with a total duration of 2.26GB.
• The audio tracks are from a variety of genres, including rock, pop, classical, and jazz.
• The audio tracks are annotated with the following information: Tempo and Meter class

## 3 Experimental Design, Materials and Methods

**Fig. 2:**
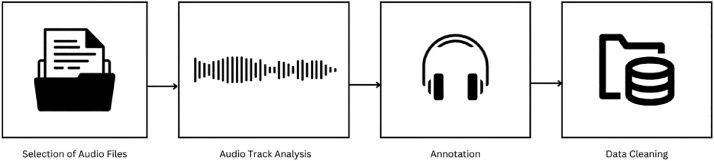
Meter2800 dataset creation process.

The dataset was created by combining three popular MIR datasets (GTZAN [2] – 1000 audio files, FMA-medium [3] with 25,000 audio files and MagnaTagATune [4] with about 23,000 audio files) and adding new data to address the deficiencies in the other three. The creation process for the Meter2800 dataset involved several steps. First, a set of audio files was selected for analysis. The files were chosen to represent a diverse range of musical genres and styles. Next, every audio track of 30 seconds duration in the individual dataset was carefully annotated by music experts. The annotators listened to each track multiple times to identify the time signature, which is the meter of the music. After identifying the time signature, the annotators recorded this information as 3, 4, 5 or 7, along with other relevant metadata, such as the musical tempo and other extracted low level features. The tempo and the extracted features were processed using librosa [5]. The extracted features are Chroma Short Time Fast Fourier Transform (STFT) [6], Root Mean Square (RMS), Mel-Frequency Cepstral Coefficients [7], Spectral Centroid [8], Spectral Bandwidth [9], Zero Crossing Rate [10] and Spectral Rolloff [11]. These features are stored in a separate csv file called multiple-features.csv. A summary of the process is shown in (Fig. 2).

The code files used to extract the audio features can be found on the github repository [12], in a folder called Meter2800. These open-source jupyter notebooks are designed to streamline the process of extracting valuable data from audio files for research and analysis purposes.

## Limitations

The dataset consists of 2800 annotated audio samples, each of which is 30 seconds in length, grouped into four meter classes. The dataset includes annotations of the time signatures for each audio track, which were determined through a manual listening process. The classes 3 and 4 of the Meter2800 dataset contain 1200 audio samples each, while classes 5 and 7 have 200 audio samples each, resulting in an uneven distribution of data. This is because the contributing datasets, such as the GTZAN, FMA, and MagnaTagATune, heavily feature the most popular musical genres such as hip-hop, rock, and pop, which are generally characterized by simple beat counts that are easy to estimate. As a result, the majority of the audio tracks in these datasets have meter numerator values of 2, 3, and 4. On the other hand, audio tracks with irregular meters are less common and more challenging to detect, which accounts for the dataset's imbalance (Table 2).

**Table 2:** Summary of annotated files by class for Meter2800 dataset.

Class	Number of annotated files
3	1200
4	1200
5	200
7	200

## Ethics Statement

In terms of distribution and reuse, the GTZAN dataset and the MagnaTagATune dataset are distributed under a Creative Commons Attribution-NonCommercial-ShareAlike 4.0 International (CC BY-NC-SA 4.0) license. This means that these datasets can be shared and adapted for non-commercial purposes, provided that proper attribution is given to the original creators, any adaptations are shared under the same license, and no commercial use is made of the datasets or any derived works. As the intended use of the datasets in this research is non-commercial, it falls within the terms of the license and can be utilized accordingly. On the other hand, the Free Music Archive (FMA) dataset is distributed under a Creative Commons Attribution 4.0 International (CC BY 4.0) license. This license allows the dataset to be shared, adapted, and used for commercial purposes, as long as appropriate attribution is given to the original creators.

Furthermore, the songs in the OWN folder of the research project are only songs under the Creative Commons CC-BY license sourced from the YouTube platform. This license enables content creators to retain their copyright while allowing other users to reuse their work, subject to the terms of the license*.*

## CRediT authorship contribution statement

**Jeremiah Abimbola:** Data curation, Methodology, Writing – original draft. **Daniel Kostrzewa:** Conceptualization, Writing – review & editing. **Paweł Kasprowski:** Writing – review & editing.

## Data Availability

METER2800 (Original data) (Dataverse).
